# Octreotide modulates the expression of somatostatin receptor subtypes in inflamed rat jejunum induced by *Cryptosporidium parvum*

**DOI:** 10.1371/journal.pone.0194058

**Published:** 2018-03-09

**Authors:** Jie Bai, Xin Liu, Le Goff Laetitia, Gargala Gilles, Francois Arnaud, Ballet Jean Jacques, Ducrotte Phillipe, Favennec Loic, Liqianhai Towledahong

**Affiliations:** 1 Department of Pharmacology, XinJiang Medical University, Urumqi, XinJiang, China; 2 Parasitology Department, Rouen University Hospital & EA 4311-IFRMP 23, Institute for Biomedical Research, University of Rouen, Rouen, France; 3 Department of Histopathology, CHU Charles Nicolle, Rouen, France; 4 Immunology Department, Caen University Hospital & UPRES-EA 2128, University of Caen, Caen, France; 5 Gastroenterology Unit, Rouen University Hospital & EA 4311-IFRMP 23, In Institute for Biomedical Research, University of Rouen, Rouen, France; Albert Einstein College of Medicine, UNITED STATES

## Abstract

Somatostatins are proteins that are involved in gastrointestinal function. However, little is known with regard to somatostatin receptor subtype (SSTR) expression changes that occur in the jejunum during low-grade inflammation and during subsequent octreotide treatment. The aim of the present study was to investigate the expression of SSTRs in the jejunums of *Cryptosporidium parvum* (*C*. *parvum*)-infected rats by immunohistochemisty, reverse transcription (RT) PCR and quantitative real-time RT-PCR assays. Five-day-old suckling Sprague-Dawley rats (n = 15 for each group) were orally gavaged with 10^5^ Nouzilly isolate (NoI) oocysts. Rats then received 50 μg/kg/day of octreotide by intraperitoneal injection from day 10 to day 17 post-infection. Animals were sacrificed on days 7 and 14 post-infection for immunohistochemical analysis and on days 14, 35 and 50 for mRNA expression analysis of SSTR subtypes. Histological analysis of jejunum tissues demonstrated infection of *C*. *parvum* along the villus brush border on day 7 post-infection and infection clearance by day 14 post-infection. Real-time PCR analysis indicated that in the inflamed jejunum, a significant increase in SSTR1 and SSTR2 expression was observed on day 14 post-infection. Octreotide therapy down-regulated the expression of SSTR2 on day 37 post-infection but significantly increased expression of SSTR1, SSTR2 and SSTR3 on day 50 post-infection. The results indicate that specific SSTRs may regulate the inflammatory pathway in the rat intestinal inflammation model.

## Introduction

Somatostatin (SOM), also known as growth hormone inhibiting hormone (GHIH), is a peptide hormone that is released in response to growth hormone secretion and interacts with the nervous, endocrine and immune systems in order to induce specific biological functions. Although somatostatin and its analogues have been shown to modulate a number of immune functions, their effects vary and are strongly dependent on the cell type in which they are expressed [1].

Previous studies have shown that the activation of nociception is sensitive to inflammatory mediators and capsaicin, resulting in pain sensation and the release of sensory neuropeptides [2,3]. Somatostatin is stored in a capsaicin-sensitive subpopulation of nociceptors, where it is released and depleted [4]. A systemic anti-inflammatory response is then elicited when sufficient amounts of somatostatin are released from activated primary afferent nerve terminals [4].

SSTR expression is increased in human intestinal inflammation, suggesting that SSTRs may be involved in the pathophysiology of inflammatory bowel disorders [5]. Thus, somatostatin may act as a potentially powerful inhibitor of a complex and auto-regulatory inflammatory cascade coordinated by mast cells and neuron bidirectional communication at the site of inflammation [6]. Additionally, somatostatin exerts a strong anti-nociceptive effect via the modulation of extrinsic afferent nerve fibers. Therefore, it is essential to evaluate the expression of SSTRs during inflammation and the use of somatostatin analogs in the treatment of inflammatory intestinal conditions.

Octreotide is a synthetic somatostatin cyclic analogue that preferentially activates SSTR2 and SSTR5. Although traditionally used to treat carcinoids and islet cell tumors [7], it further exhibits optimal efficacy in the treatment of certain pain conditions. The beneficial effects of octreotide are mainly attributed to its direct inhibition of hormone production, decrease in intestinal fluid production, and decrease in intestinal contractility.

Previous studies demonstrated that in a suckling immunocompetent rat model, *Cryptosporidium parvum* (*C*. *parvum*) infection induced jejunal hypersensitivity to distension [8]. This effect lasted more than 100 days following the spontaneous clearance of parasites [8]. It has further been shown that the antinociceptive and antiinflammatory actions of octreotide are largely mediated via the SSTR2 receptor, whereas the SSTR1 receptor is considered a novel pharmacological target for somatostatin-mediated peripheral analgesia in inflammatory pain [9]. Mechanistically, octreotide has been shown to stimulate NHE8 expression in colitic mice [10]. NHE8 is inhibited during colitis, and SST treatment during pathological conditions can restore NHE8 expression [10]. Based on these studies, we developed a hypothesis that the immune cell-derived somatostatin may contribute to systemic immunomodulatory effects that extend beyond its known anti-nociceptive and anti-inflammatory effects. Thus, the aim of the present study was to investigate the effect of *C*. *parvum* infection on the expression patterns of SSTR1, SSTR2 and SSTR3 subtypes in the rat jejunum. In addition, the short-term and long-term effects of the somatostatin analogue, octreotide, on the SSTR subtype expression were examined.

## Materials and methods

### Ethics statement

The research with title: “Octreotide modulates the expression of somatostatin receptor subtypes in inflamed rat jejunum induced by *C*. *parvum*” has obtained research ethics approval from the Institutional Care and Use committee (IACUC) of the Xinjiang Medical University for the use of oocysts and the experimental protocol that involved infection of rats, sacrifice and collection of .jejunum, ileum and colon tissue samples for subsequent experimentation. The IACUC, and/or competent authority, has granted approval for this method, provided adequate training for the technique is provided, and its continued approval is re-evaluated as more scientifically-based data regarding its use become available. The protocol was in accordance with the European Communities Council Directive of 24 November 1986 (86/609/EEC). Animals were sacrificed prior to all tissue extractions.

### C. parvum oocysts

Oocysts from the Nouzilly isolate (NoI) were a kind gift from R. Mancassola and M. Naciri (Laboratoire de Pathologie Aviaire, Institut National de Recherche Agronomique, Nouzilly, France). Oocysts were maintained and purified as previously described [11]. Briefly, feces from experimentally infected calves were obtained and stored in a 2.5% K_2_Cr_2_O_7_ solution for a total period of 3 months. Oocysts were rinsed in phosphate-buffered saline (PBS) prior to infection [12].

### Infection of unweaned rats

*C*. *Parvum* pathogenicity was evaluated using five-day-old suckling Sprague-Dawley rats (Janvier, Le Genest Saint Isle, France) as previously described [13]. Dams and their litters were maintained free of *C*. *parvum* infection. Rats were maintained in specific pathogen-free conditions that included separate housing in plastic cages and the administration of heat-sterilized food and water *ad libitum*. Suckling rats received an oral gavage of 100 μl PBS containing 10^5^ NoI oocysts and/or 100 μl PBS alone for control rats. Preliminary experiments demonstrated a maximum ileal burden between day 6 and day 8 post-infection (PI). The onset of parasite clearance was noted following 14 days of infection [14]. Sprague-Dawley rats were divided into four treatment groups: 1) control, which were non-infected and untreated group (n = 15); 2) non-infected group, treated group (n = 15); 3) infected, untreated group (n = 25); 4) and infected, treated group (n = 15). The control group is mainly limited to the 7 day period of infection in order to establish the absence of infection. Accordingly, the octreotide treatment corresponds to a 7 day period from day 10 to day 17. The specific time points of the control groups were adapted from the experimental protocol of a previous study [13].

### Octreotide treatment

Octreotide was purchased from Sigma-Aldrich (St. Louis, Missouri, USA) and resuspended in water for animal gavage. Animals received 50 μg/kg/day of octreotide treatment. The compound was administrated by intraperitoneal injection from day 10 PI to day 17 PI. Both uninfected rats and rats infected with 10^5^ oocysts received octreotide treatment.

### Immunohistochemistry

On days 7 and 14 PI, rats were sacrificed for histological and cytological examination. Jejunum, ileum and colon tissue samples were collected, fixed in 10% formalin, embedded in paraffin and cut into 5 mm-thick sections. The tissues were stained with Giemsa in order to evaluate the infection by *C*.*parvum*. *C*.*parvum* mucosal formations in the jejunum and ileum were counted in each section in order to estimate the parasite burden. A total of 10 well-oriented villus-crypt units (VCU) were counted. The parasite burden was expressed as the number of parasites per millimeter of villus [8]. For PGP 9.5 immunoreactivity a similar experimental protocol was used as previously described [15]. Briefly, the tissue sections were subjected to heat mediated antigen retrieval using citric acid. The sections were then blocked using 1% BSA for 10 min at 21°C. Primary antibody against PGP 9.5 (ab8189) was then incubated for 16 hr at 21°C at 1:1,000 dilution. The secondary antibody used was Goat polyclonal to anti mouse IgG conjugated to biotin that was purchased from Abcam (CA, UK) (1:200). The secondary antibody was incubated for 15 min at room temperature and detected using an HRP conjugated compact polymer system (Abcam, CA, UK).

### Quantitative real-time PCR

Rats from each treatment group were sacrified on days 14, 37 and 50 PI. Tissues from the distal jejunum were extracted for RNA isolation. The tissues were immediately frozen in liquid nitrogen and stored at -80°C. A sample of brain cortex tissue from a non-infected rat was frozen in liquid nitrogen and used as a positive control for reverse transcription and quantitative real-time PCR. Jejunum RNA was extracted using Trizol reagent (Sigma-Aldrich, Saint-Quentin Fallavier, France) according to the manufacturer’s instructions. Reverse transcription was conducted using 2 μg of RNA and the High-Capacity cDNA reverse transcription kit (Applied Biosystems, Courtaboeuf, France) according to the manufacturer’s instructions. All primer sequences used for the detection of SSTR1, SSTR2 and SSTR3 genes have been described previously [16] and are listed in Table 1. All reactions were carried out using the Lightcycler FastStart DNA MasterPLUS SYBR Green I kit (Roche Diagnostic, Meylan, France) in a reaction volume of 20 μl. The samples were subjected to 45 cycles of amplification. Each cycle consisted of a denaturation step at 95°C for 15 sec, an annealing step at 61, 61 and 63°C for 5, 10 and 4 sec and an extension step at 72°C for 2, 15 and 10 sec with regard to the amplification of SSTR1, SSTR2 and SSTR3 sequences, respectively. A melting curve analysis was used to determine the specificity of the amplified products. All reactions were conducted in duplicate.

**Table 1:** Real time PCR primer parameters including sequences, product length and melting point temperature.

Gene	Strand	Primer sequence	Position	Product length	Tm
SSTR1	Sense	5’-TACTACGCCACTGCCCTGAAG-3’	1201–1221	54 bp	84°C
	Antisense	5’-AGGCTGGAAGTCCTCCACACT-3’	1234–1254		
SSTR2	Sense	5’-GGGAGCCAAGTGTGGATACCT-3’	395–415	107 bp	88.5°C
	Antisense	5’-ACCGCGTTGCTTGTCATGT-3’	483–501		
SSTR3	Sense	5’-ATGGCCGCTGTTACCTATCCT-3’	656–676	111 bp	87°C
	Antisense	5’-GCTAGTGCCAGCAGATGCATT-3’	746–766		
β-actin	Sense	5’-CTGCCCTGGCTCCTAGCA-3’	753–770	52 bp	85.5°C
	Antisense	5’-CGCTCAGGAGGAGCAATGA-3’	786–804		

### Statistical analysis

Data were expressed as the mean ± standard deviation (SEM: 95% confidence intervals assuming normal distribution of the data). The assessment of the normality of the data was conducted using the Kolmogorov Smirnov test. A Student’s *t* test was used to compare the non-infected and infected groups. The Mann-Whitney U test was used to evaluate significant differences between groups for comparisons of 2 groups when the data did not follow a normal distribution. For categorical variables the chi-squared test was used. A P value of less than 0.05 (P <0.05) was considered significant.

## Results

### Mucosal alterations in infected animals

**Fig 1:**
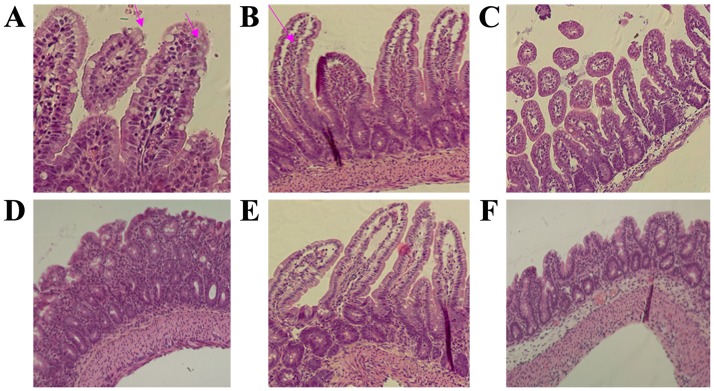
Representative immunohistochemical images of alterations in jejunal, ileal and colonic mucosae following *C*. *parvum* infection. A, B and C represent jejunum, ileum and colon tissues, respectively, following 7 days ofinfection. The red arrows indicate typical parasite formations. D, E and F represent jejunum, ileum and colon tissues, respectively, following 14 days of infection. Magnification x 400. *C*. *parvum*, *Cryptosporidium parvum*.

**Fig 2:**
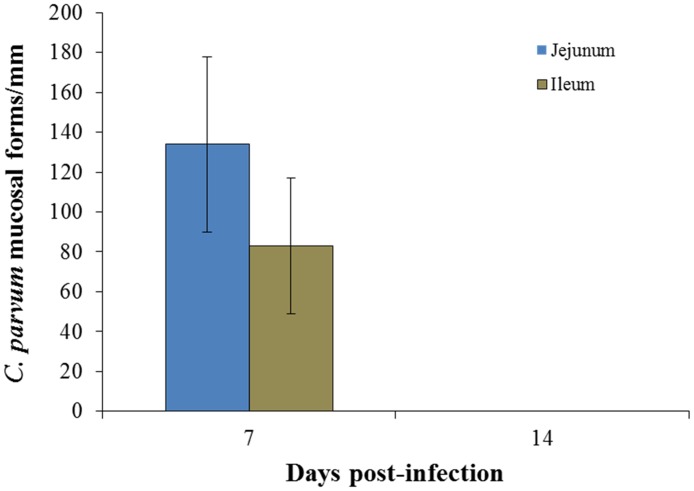
Parasite formation counts in jejunal and ileal mucosae from *C*. *parvum*-infected animals. Four-micrometer sections were stained with Giemsa and considered infected if at least one cryptosporidial developmental form was observed within one mucosal cell, as determined previously [13]. At least 20 fields were counted that contained the highest number of parasites. The values represent the mean ± standard deviation (n = 5 in each group). *C*. *parvum*, *Cryptosporidium parvum*.

The parasite burden was evaluated based on our previous findings and the technique used was based on calculation by counting the *C*. *parvum* mucosal forms in the ileum in each section on 10 well-oriented villus-crypt units (VCU) [8]. The parasite burden is expressed as the number of parasites per millimeter of villus [8]. As shown in Fig 1, *C*. *parvum* was detected on the outside villus brush border of the jejunum of infected rats at the peak of infection on day 7 PI. Concomitantly, villi atrophy and crypt hyperplasia were observed. The parasite counts were significantly higher in the jejunum mucosa compared with the ileum mucosa of the infected rats (Fig 2). Following self-clearance of oocysts on day 14 post-infection *C*. *parvum* could not be detected in the jejunum, ileum and colon as demonstrated by histological evaluation (Fig 2).

### Evaluation of jejunal somatostatin receptor subtype mRNA levels in *C*. *parvum-infected* and non-infected rats

**Fig 3:**
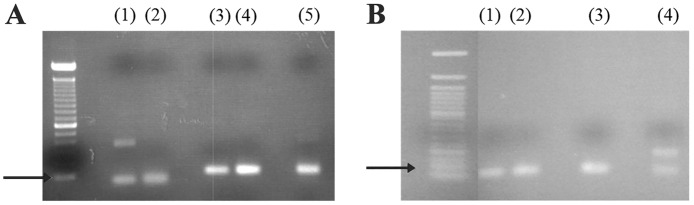
Representative gel agarose images of SSTR PCR products following q-PCR analysis. A, A 2% agarose gel was stained with ethidium bromide following qPCR to verify the detection of the gene products. (MW) 100 bp DNA ladder, (1) brain SSTR1, 54 bp, (2) jejunum SSTR1, 54 bp, (3) brain SSTR2, 107 bp, (4) jejunum SSTR2, 107, bp, (5) jejunum SSTR3, 111 bp. B, A 1.8% agarose gel was stained with ethidium bromide following qPCR to verify the detection of the gene products. (MW) 50 bp DNA ladder, (1) jejunum β-actin, 52 bp, (2) brain β-actin, 52 bp, (3) jejunum SSTR1, 54 bp, (4) brain SSTR1, 54 bp. Black arrows indicate the 100 bp marker.

SSTR1, SSTR2 and SSTR3 expression levels were detected in jejunal tissue samples using quantitative real-time PCR. Fig 3 indicates representative PCR amplification products of SSTR1, SSTR2 and SSTR3 cDNA sequences. No DNA contamination was detected as demonstrated by the lack of amplification products using non-reverse transcribed mRNA control samples. The melting curve analysis demonstrated that each reaction of the SSTR PCR products was conducted at an optimal melting temperature (Tm) (Table 1) and the product sizes were consistent with those noted by agarose gel electrophoresis (Fig 3A and 3B). The sequencing results of the amplified RT-PCR products from SSTR1, SSTR2 and SSTR3 indicated that the homology was 100%, 98% and 99%, respectively (S1 Table).

### Sequential quantification of jejunal somatostatin receptor subtype mRNAs in *C*. *parvum-infected* and non-infected rats

**Fig 4:**
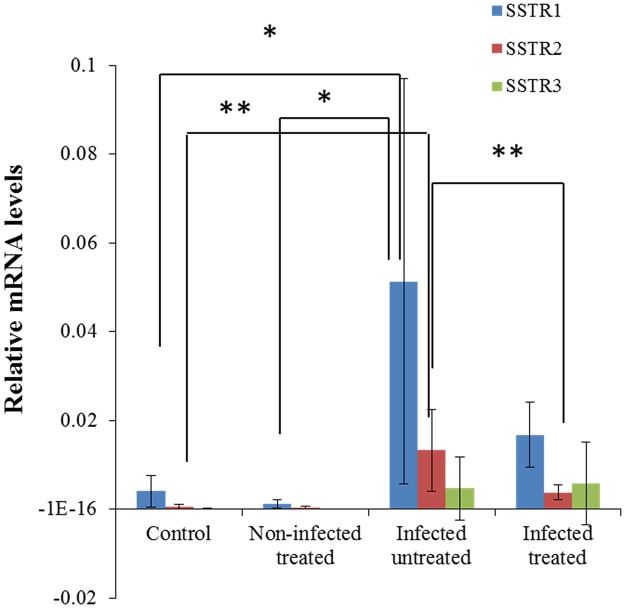
Sequential modulation of SSTR1, SSTR2 and SSTR3 jejunal mRNA levels in *C*. *parvum*-infected or octreotide-treated rats on day 14 post-infection. The bar chart represents the quantification of the PCR results that corresponds to the mRNA levels of expression of SSTR genes. The values represent mean ± standard deviation (n = 5 in each group). *P<0.05, **P<0.01, infected, untreated vs. control and infected, untreated vs. infected, treated. *C*. *parvum*, *Cryptosporidium parvum*.

The levels of jejunal SSTR1, SSTR2 and SSTR3 mRNA transcripts increased by more than 98% from day 14 to day 37 post-infection (Fig 4). However, the transcript levels were decreased by at least 70% from day 42 to day 50 post-infection in the control animals. Following the period of intestinal parasite clearance, by day 14 post-infection, the mRNA levels of SSTR1, SSTR2, but not SSTR3 were significantly higher in the tissues extracted from the jejunum of *C*. *parvum*-infected animals compared with those of the control animals (Fig 4, p<0.05).

**Fig 5:**
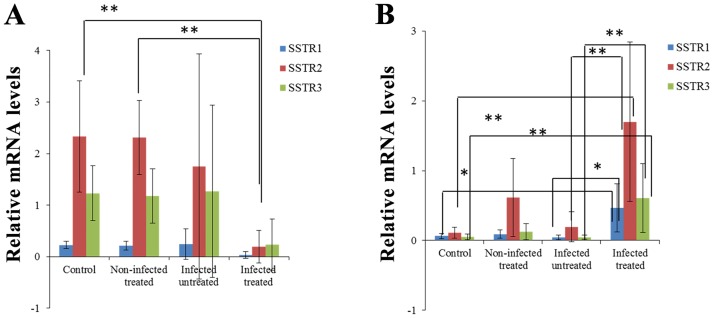
Sequential modulation of SSTR1, SSTR2 and SSTR3 jejunal mRNA levels in *C*. *parvum*-infected or octreotide-treated rats on A. day 37 post-infection B. day 50 post-infection. The bar charts represent the quantification of the PCR results that correspond to the mRNA levels of expression of SSTR genes. The values represent mean ± standard deviation (n = 5 in each group). *P<0.05, **P<0.01, infected, untreated vs. control and infected, untreated vs infected, treated. *C*. *parvum*, *Cryptosporidium parvum*.

Octreotide therapy significantly reduced SSTR1 and SSTR2 mRNA levels compared with the infected, untreated control group (p<0.01). SSTR1 and SSTR3 mRNA transcript levels were not significantly increased in previously infected, untreated animals on day 37 post-infection (Fig 5A) compared to the infected, treated and non-infected control groups. On day 50 post-infection, previously infected, octreotide-treated animals exhibited significantly elevated levels of SSTR1, SSTR2 and SSTR3 mRNA compared with previously infected, untreated rats (p<0.05 and p<0.01, respectively, Fig 5B).

**Fig 6:**
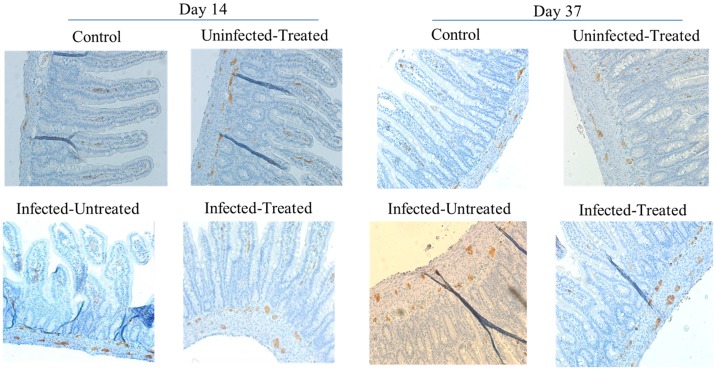
PGP 9,5 immunoreactivity in rat jejunum infected by *C*.*parvum* on days 14 and 37 post-infection. Protein gene product 9.5 (PGP 9.5) is a marker for neuroendocrine cells. The immunohistochemistry of routinely processed neuronal tissues was conducted in tissues that were obtained at days 14 and 37 for the 4 different groups, namely infected, control, infected untreated and infected treated.

Following 14 days of infection, the density of jejunal nerve fibres was increased in infected untreated rats compared with uninfected untreated rats both in the submucous plexus and myenteric plexus areas, but not in the villous tissue, as demonstrated by PGP9,5 immunoreactivity, (Fig 6, Table 2). Octreotide therapy decreased jejunal nerve fibres both in the uninfected and infected rats. On day 37 post-infection, the density of jejunal nerve fibres increased in the submucous plexus and villous areas, but not in the myenteric plexus tissues in infected untreated rats compared with uninfected untreated rats (Fig 6, Table 2). This trend of expression persisted until day 50 post-infection (Table 2).

**Table 2:** The density of the jejunal nerve fibres in the submucous plexus myenteric plexus areas and villous tissue. The mucosal formations in the jejunum and ileum were counted by PGP 9.5 immunoreactivity in each section in order to estimate the parasite burden. A total of 10 well-oriented villus-crypt units (VCU) were counted. The parasite burden was expressed as the number of parasites per millimeter of villus. For PGP 9.5 immunoreactivity a similar experimental protocol was used as previously described in the materials and methods section. A total of 3 time periods were used namely, day 14, 37 and 50 post-infection.

		D14 P.I			D37 P.I			D50 P.I	
	PGP9,5/ submucous plexus (um^2^/um^2^)	PGP9,5/ myenteric plexus (um^2^/um^2^)	PGP9,5/ villous (um^2^/um^2^)	PGP9,5/ submucous plexus (um^2^/um^2^)	PGP9,5/ myenteric plexus (um^2^/um^2^)	PGP9,5/ villous (um^2^/um^2^)	PGP9,5/ submucous plexus (um^2^/um^2^)	PGP9,5/ myenteric plexus (um^2^/um^2^)	PGP9,5/ villous (um^2^/um^2^)
Uninfected + untreated	3,00±0,90	3,50±1,17	1,21±0,97	2,28±0,62	4,03±2,03	0,50±0,14	0,70±0,50	0,33±0,20	0,62±0,05
Uninfected + treated	2,76±1,15	2,98±1,84	0,70±0,66	2,58±1,96	3,60±1,49	0,42±0,16	1,07±0,44	0,29±0,19	0,96±0,24
Infected + untreated	4,15±1,72	4,15±1,78	1,12±0,55	3,14±0,65*	3,52±0,91	0,69±0,42	1,43±0,61*	0,64±0,41*	1,23±0,33*
Infected + treated	3,15±0,42	3,18±2,55	0,50±0,38^Δ^	2,50±1,53	2,83±1,22	0,41±0,24	0,74±0,58^Δ^	0,06±0,05^ΔΔ^	0,68±0,32^Δ^

Compared with uninfected untreated rats,

*P<0.05;

Compared with infected untreated rats,

^Δ^P<0.05,

^ΔΔ^P<0.01

## Discussion

Previous studies have provided evidence regarding the immunomodulatory role of somatostatin in the inflamed intestine. The present study adds further insight in the putative mechanism of action of octreotide with regard to the regulation of the expression of SSTR1, SSTR2 and SSTR3 receptors. The histological characteristics of the infected jejunum were in accordance with the previously described study [8]. The quantitative PCR results demonstrated that on day 14 post-infection, SSTR1, SSTR2 and SSTR3 mRNA comprised approximately 85%, 12.5%, and 2.5% of SSTR subtypes, respectively in the unweaned rat model. This is in line with a previous study that demonstrated SSTR1 and SSTR3 expression by *in situ* hybridizaton with riboprobes [17]. The expression of the subtypes SSTR1 and SSTR2 has been documented in rats in different disease models, such as the epilepsy model [18]. The data indicated that on day 14 post-infection, SSTR1 and SSTR2 mRNA levels were significantly increased. The development of intestinal cryptosporidiosis in this rat model and the increased somatostatin mRNA levels suggest an immunomodulatory role for SSTRs in the rat jejunum under normal and infectious conditions. These results are in accordance with the studies using *Schistosoma mansoni* (*S*. *mansoni*) -infected SSTR2 null and wild-type mice [19].

Although SSTR1 and SSTR2 expression levels were increased following infection, SSTR3 mRNA was not significantly modified on days 14, 37 and 50 post-infection. These results may be due to the low concentration of SSTR3 mRNA expressed in the jejunum. The data are also inconsistent with a previous study that examined SSTR3 expression in the ileum of *S*. *mansoni*-infected mice eight weeks post-infection [20]. Further studies using more sensitive methods of mRNA and protein detection are required to elucidate the exact role of SSTR3 in inflammation.

The present study demonstrated that on days 37 and 50 post-infection, the parameters number of mast cells in the lamina propia, number of intraepithelial lymphocytes and nerve fiber density were elevated in the submucous and myenteric plexuses in the jejunum of rats infected by *C*. *parvum*. On day 100 post-infection, jejunal hypersensibity to distension also appeared. These results suggested that SSTR1 and SSTR2 mRNA levels increased earlier in immunocytes and neurons compared with the jejunum, whereas a role for SSTR1 and SSTR2 in the immune response to intestinal infection was demonstrated.

SSTRs regulate diverse physiological actions in the gastrointestinal tract, including the inhibition of endocrine and exocrine secretions, gut motility and the proliferation of lymphocytes and mast cells. Previous studies have reported the potent inhibitory effects of somatostatin on mucus mast cell degranulation in the rat intestine, possibly by direct activation of SSTR1. SSTR2A expression has been noted in murine enteric glial cells [20]. This study provided evidence that enteric glial cell activation and subsequent release of inflammatory mediators play an important role in intestinal infection [21]. Activation of SSTR2 in peripheral primary afferent nerves diminishes spontaneous nociceptive behavior and reduces the response to excited nociceptors.

The present study demonstrated that octreotide therapy, administered at 50 μg/kg/day by intraperitoneal injection from day 10 to 17 post-infection, decreased mRNA levels of SSTR1 and SSTR2 on day 14 post-infection, although it increased mRNA levels of SSTR1, SSTR2 and SSTR3 on day 50 post-infection. It may be speculated that the increased SSTR mRNA levels may result in a reduced number of mast cells and intraepithelial lymphocytes by day 50 and a consequent hypersensitivity to jejunal distension by day 120 post-infection. Similar studies have demonstrated loss of the beneficial therapeutic effect of octreotide following an extended period of time (several months), presumably due to receptor desensitization, SSTR structural changes and/or loss of SSTR expression [22]. Although the half-life of octreotide is only 90–100 min, its effects could last longer than 30 days, possibly due to the SSTR-mediated immnunomodulatory and anti-inflammatory action. The induction of the expression levels of SSTR has been associated with the induction of inflammation, whereas antinociceptive and antiinflammatory actions of octreotide and pasireotide were largely mediated via the SSTR2 receptor [23]. The downregulation of SSTR1, 2 and 3 expression noted in the octreotide treated infected rats on days 14 and 37, is possibly a secondary effect following the reduction of inflammation and thus this is not solely dependent on the half life of the drug.

Loss of treatment efficacy may be caused by upregulation of the receptor and/or interaction between SSTR subtypes. Many G-protein coupled receptors exhibit receptor desensitization due to uncoupling from G proteins, receptor internalization and receptor degradation [22]. It has been shown that SSTR1, SSTR2, SSTR3 and SSTR5 exhibit acute desensitization of adenylate-cyclase coupling [24–27].

Cryptosporidium infection can cause increased 5-day-old jejunum sensitivity in suckling mice until 100 days after the infection [28]. Octreotide reduced jejunal hypersensitivity in a rat model [29]. In addition, octreotide can reduce pain [6]. To explore the underlying mechanism, we detected the submucosal nerve plexus of the jejunal tissue, intestinal intramuscular plexus and villus nerve fibers post Cryptosporidium infection (The nerve cells were labeled in 5 μm sections with a monoclonal mouse PGP 9.5 antibody). In addition, it has been shown that SSTR1 is upregulated in a rat intracerebral hemorrhage (ICH) model, whereas the SSTR1 protein was mostly co-localized with neurons, and was rarely distributed in activated astrocytes and microglia [30]. Furthermore, SSTR1 co-localized with active-caspase-3 and bcl-2 around the hematoma, while the expression of active-caspase-3 was parallel with that of SSTR1 in a time-dependent manner, indicating that up-regulated SSTR1 contributed to neuronal apoptosis following ICH [30]. These findings are in agreement with the data presented from the PGP 9.5 immunoreactivity assay, suggesting that SSTR expression may be associated with neuronal apoptosis and consequently decrease in the number of jejunal nerve fibres both in the uninfected and the infected rats.

In conclusion, *C*. *parvum* infection modulated the expression of different SSTR subtypes in a rat model. The results suggested an immunomodulatory role of SSTRs in the rat jejunum in the healthy state and under infectious conditions. We speculate that downstream effects may include interaction with activated mast cells, lymphocytes, submucous plexus and somatostatin. Future studies using dual labeling immunocytochemical techniques are required to further elucidate the roles of SSTRs in inflammation.

## Supporting information

S1 File Animal ethics certification. (PDF)

S1 Table Sequencing results of the RT-PCR products derived from SSTR1, SSTR2 and SSTR3 fragment amplification. (DOCX)
