# Correction: Octreotide modulates the expression of somatostatin receptor subtypes in inflamed rat jejunum induced by *Cryptosporidium parvum*

**DOI:** 10.1371/journal.pone.0198377

**Published:** 2018-05-24

**Authors:** Jie Bai, Xin Liu, Laetitia Le Goff, Gilles Gargala, Arnaud François, Jean Jacques Ballet, Phillipe Ducrotte, Loic Favennec, Liqianhai Towledahong

The third, fourth, fifth, sixth, seventh, and eighth authors’ names are incorrect. The correct author list is: Jie Bai, Xin Liu, Laetitia Le Goff, Gilles Gargala, Arnaud François, Jean Jacques Ballet, Phillipe Ducrotte, Loic Favennec, Liqianhai Towledahong. The correct citation is: Bai J, Liu X, Le Goff L, Gargala G, François A, Ballet JJ, et al. (2018) Octreotide modulates the expression of somatostatin receptor subtypes in inflamed rat jejunum induced by *Cryptosporidium parvum*. PLoS ONE 13(3): e0194058. https://doi.org/10.1371/journal.pone.0194058.
