# Supplementary material for: Octreotide modulates the expression of somatostatin receptor subtypes in inflamed rat jejunum induced by Cryptosporidium parvum
Source: PLoS One. 2018 Mar 9;13(3):e0194058. doi: 10.1371/journal.pone.0194058 (PMC5844672; doi:10.1371/journal.pone.0194058)
Supplement: S1 File — (PDF) [file pone.0194058.s001.PDF]

A-3（2.0版）

新疆医科大学第一附属医院动物实验医学伦理委员会审批报告

伦理审批号：IACUC20160616-06

审查日期	2016-06-16	审查会议地点	科技楼 327		
研究项目名称	奥曲肽改善肠易激综合症的机制研究				
研究科室	新疆医科大学基础医学院	主要研究者	白杰		
送审文件	实验动物使用和管理（IACUC）准入申请表				
审查状态	■初审　□复审	项目来源	国家级自然科学基金		
实验起止时间	2016.6-2016.8				
主审委员	姜涛				
审查结果及意见	投票人数	准入预实验	准入正式实验	暂停实验	终止实验
	8		8		
	实验动物使用和管理委员会对本课题预实验的结果及《兽医评估报告》的有关内容进行了会议审查，审查结果为“同意”经伦理委员会委员审查认为该研究符合伦理原则，**同意开展正式实验。**<br><br>主任或副主任委员签字：（签名）<br>医学伦理委员会（盖章）<br>日期：2016 年 6 月 28 日				

附件：伦理委员会名单签到及保密协议

抄送：1. 科研科

2. 实验研究人员：　　　　日期：

3. 研究者签收：　　　　日期：

The Medical Ethics Committee of First Affiliated Hospital to XinJiang Medical University for experiments on animals approval certification

Number of Ethics approval: IACUC20160616-06

Date of investigate: 2016-06-16

Site of investigate meeting: Room 327 Science and Technology Building

Research Project: Effect of Octreotide on post-infection irratable bowel syndrome induced by *Cryptosporidium parvum* (No.81360071)

Research Division: Preclinical Medicine College, XinJiang Medical University

Researcher: Jie BAI

Documents for submit: Institutional Animal Care and Use Committee (IACUC) Application Form

Investigate state: First trial

Project resource: National Natural Science Foundation

Trails beginning and ending time: 2016.06---2016.08

Chief Umpire:Tao JIANG

Investigate Judgement and Results

Number of people to Vote: 8

Number of people for approval: 8

After investigating preliminary experimental results for this project and related contents of《Veterinarian Evaluation Reports》, Institutional Animal Care and Use Committee think this research are in accordance with animal ethic standards and all agreed to perform formal experiments.

The results of investigation are: "AGREE".

Director signature [illegible]

Institutional Animal Care and Use Committee (seal)

Date: 2016. 6. 28

Attachment documents: sign in sheet and confidential agreements of Institutional Animal Care and Use Committee

To send it to: 1. Scientific research division

2. Experimental researchers

3. Researchers sign in
