# Supplementary material for: Octreotide modulates the expression of somatostatin receptor subtypes in inflamed rat jejunum induced by Cryptosporidium parvum
Source: PLoS One. 2018 Mar 9;13(3):e0194058. doi: 10.1371/journal.pone.0194058 (PMC5844672; doi:10.1371/journal.pone.0194058)
Supplement: S1 Table — (DOCX) [file pone.0194058.s002.docx]

**S1 Table** Sequencing results of the RT-PCR products derived from SSTR1, SSTR2 and SSTR3 fragment amplification

| **SSTR1** amplified cDNA: |
| --- |
| ATTCTTCTTT GCTC_GACGT ATGTCACATG AGGTGACGGA AGAGGACAGG GCAGTGGAGA C_GG_GGCTC TGTTCAATAT AGAACTTTTT CCTGCTGCTT TGGCAGCTGC CCCTTGCTCA CTCCCTGGCT CTGTGAAAGT GCTCCTTTGA CTGAAAGCCA  TCACTTCAAG TTCAGCTGGC ATGAACAGTT GT_A_ _G_C_  T_ _ _C_ _GT_ _ _ _ A_T_ _ _ _ C_T_C_ _ _ _ _ AAGAACCGGT  T_CC_GGAAT TCC_GGCT_ _ T_G_ _ _AA_ AA_A_AGGG_  TGG_ _T_ _ _G G_ _ _ _ _G_ _T _ _ _ |
| ATTCTTCTTG CTC_GACGTA TGTCACATGA GGTGACGGAA GAGGACAGGG CAGTGGAGAC _GG_GGCTCT GTTCAATATA  GAACTTTTTC CTGCTGCTTT GGCAGCTGCC CCTTGCTCAC TCCCTGGCTC TGTGAAAGTG CTCCTTTGAC TGAAAGCCAT  CACTTCAAGT TCAGCTGGCA TGAACAGTTG T_A_ _G_C_T  _ _ _ C_ _GT_ _ _ _A_T_ _ _ _C _T_C_ _ _ _ _A AGAACCGGTT  _CC_GGAATT CC_GGCT_ _T _G_ _ _AA_AA _A_AGGG_TG G_  _T_ _ _GG_ _ _ _ _ G_ _ T_ _ _ |
| Genebank (mRNA) NM_012719 |
| TACTACGCCA CTGCCCTGAA GAGTCGTGCC TACAGTGTGG  AGGACTTCCA GCCT |
| **SSTR2** amplified cDNA: |
| TC_ _ C_G_AT CA_ _AAGCC_ T_TCAACTGA C_TGCACAAG  CA_ _G_TCTT T_C_ _ _TA_A G_C_C_ _C  CTGA_TTGCA TGAGCATTGC TTGGCCCAGT GAGCCGTTGA  GGTCAAGGGA GAAGGTATCC ACACTTGGCT CA_A_G |
| Genebank (mRNA) NM_019348 |
| GGGAGC CAAGTGTGGA TACCTTCTCC CTTTGACCTC AACGGCTCAC TGGGGCCAAG CAATGGCTCC AACCAGACAG AGCCATACTA CGACATGACA AGCAACGCGG |
| **SSTR3** amplified cDNA: |
| TAAC_G_T_A TAACTT_GAC TGGGATGCAT CCTCAGCCTG  GCCCCTGGAC ACGTCCCTGG GGAATGCATC TGCTG_CACT  AG_ |
| CAGTT_AGGG GCAGGCTGAG GATGCATTCC CAGGGTCCAG GTCGTAGGCA CGGATGAAGG ATAGGTAACA GCGGCA |
| Genebank (mRNA) NM_133522 |
| ATGGC CGCTGTTACC TATCCTTCAT CCGTGCCTAC  GACCTTGGAC CCTGGGAATG CATCCTCAGC CTGGCCCCTG  GACACGTCCC TGGGGAATGC ATCTGCTGGC ACTAGC |
